# The safety and feasibility of a novel cap-assisted endoscopic resection device for rectal tissue resection: a pilot study (with videos)

**DOI:** 10.1093/gastro/goaf003

**Published:** 2025-01-30

**Authors:** Xuelian Gao, Jing Wang, Jiaming He, Panpan Liu, Haiyan Hu, Jun He, Side Liu, Yue Li

**Affiliations:** Department of Gastroenterology, Nanfang Hospital, Southern Medical University, Guangzhou, Guangdong, P. R. China; State Key Laboratory of Organ Failure Research, Key Laboratory of Infectious Diseases Research in South China, Ministry of Education, Guangdong Provincial Key Laboratory of Viral Hepatitis Research, Guangdong Provincial Clinical Research Center for Viral Hepatitis, Guangdong Institute of Hepatology, Department of Infectious Diseases, Nanfang Hospital, Southern Medical University, Guangzhou, Guangdong, P. R. China; Department of Pathology, The First Affiliated Hospital, Guangzhou Medical University, Guangzhou, Guangdong, P. R. China; Department of Gastroenterology, Nanfang Hospital, Southern Medical University, Guangzhou, Guangdong, P. R. China; Department of Gastroenterology, Nanfang Hospital, Southern Medical University, Guangzhou, Guangdong, P. R. China; Department of Gastroenterology, Nanfang Hospital, Southern Medical University, Guangzhou, Guangdong, P. R. China; Research and Development Department, Vedkang Medical, Changzhou, Jiangsu, P. R. China; Department of Gastroenterology, Nanfang Hospital, Southern Medical University, Guangzhou, Guangdong, P. R. China; Department of Endoscopy, Guangdong Provincial People's Hospital (Guangdong Academy of Medical Sciences), Southern Medical University, Guangzhou, Guangdong, P. R. China

## Introduction

In the previous study, we proved that modified cap-assisted endoscopic mucosal resection (mEMR-C) is non-inferior to endoscopic submucosal dissection for rectal neuroendocrine tumors (NETs) smaller than 10 mm with a similar complete resection rate but shorter procedure time and lower hospitalization costs [1]. However, a snare needs to be precisely placed into the inner groove of the cap during the mEMR-C procedure, which is time-consuming and difficult for inexperienced doctors. To solve this problem, we previously proposed a novel cap-assisted endoscopic resection device (CERD) for the resection of rectal lesions and initially explored the feasibility of the CERD for rectal tissue resection in an *in vitro* pig model [2]. However, the safety and feasibility of CERD for rectal tissue resection *in vivo* remain unclear.

In this present study, we modified the CERD design and aimed to evaluate the safety and feasibility of CERD for rectal tissue resection *in vivo* in pigs.

## Methods

### Experiment animals

Five 12-month-old male live pigs with a body weight of between 36 and 42 kg were randomly labeled as Pigs 1, 2, 3, 4, and 5, respectively. All pigs were fasted for 1 day before the experiments. Intramuscular zoletil (2 mL) was used to induce general anesthesia, which was maintained by using isoflurane during the experiment. All the pigs were fed with normal diet after the procedure. On the post-procedural 8th day, endoscopy was conducted to evaluate the wound condition. Institutional review board approval was obtained from the local animal ethics committee (No. BSYXIA004).

### Novel device and operation procedure

We modified the CERD based on the previous design that we reported [2]. The modified version of CERD comprises not only a transparent cap with a built-in metal snare integrated with a metal internal threaded connection part, but also a cutting device with a metal external thread connection part (Supplementary Figure 1).

**Figure 1.**
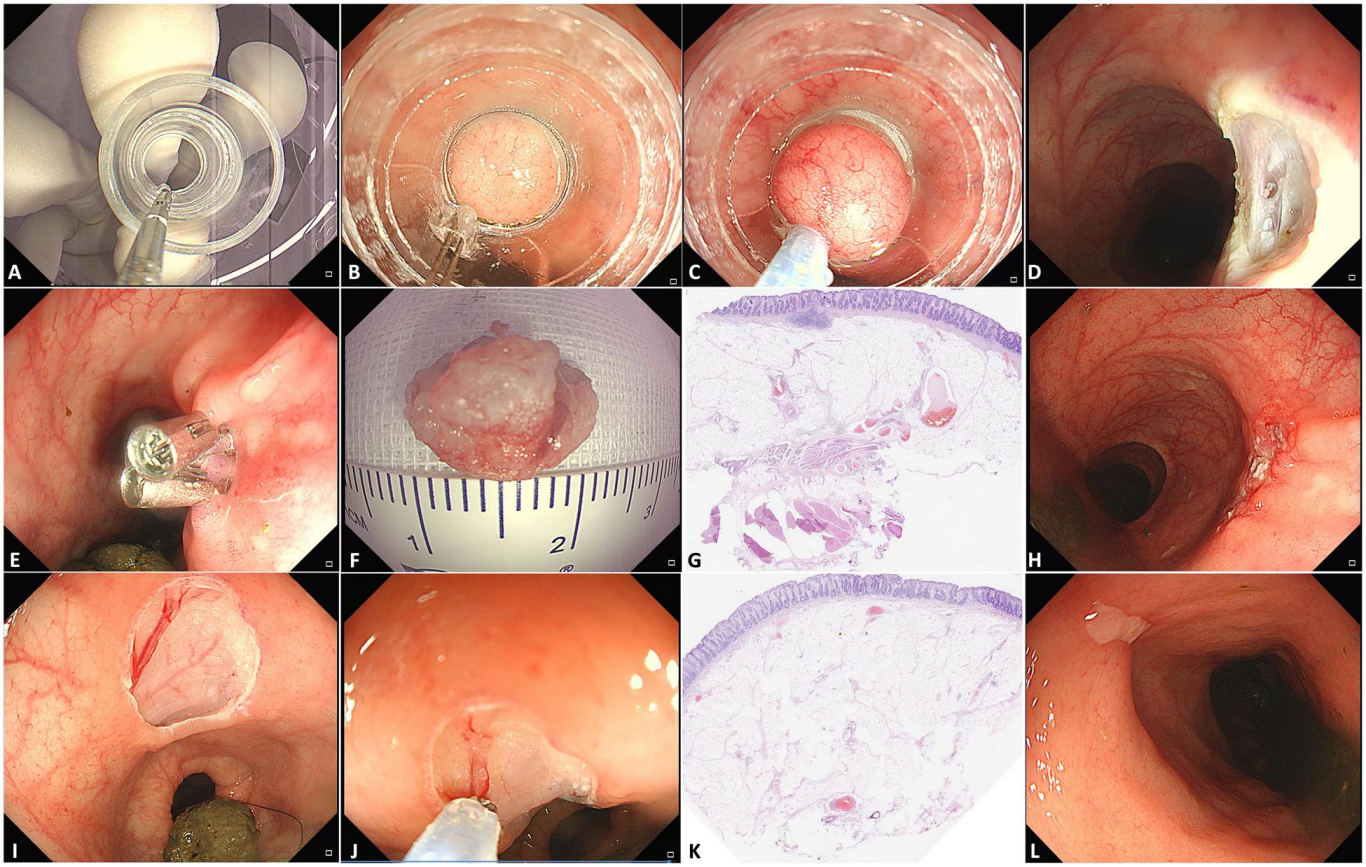
Cap-assisted endoscopic resection device-assisted rectal tissue resection in Pigs 1 and 5. (A) Installation of the transparent cap. (B) The endoscope was inserted into the rectum of Pig 1. (C) Normal rectal tissue was suctioned into the cap and the snare was tightened and closed. (D) The rectal wound after resection. (E) The wound was closed by using endoscopic clips. (F) The resected specimen was retrieved. (G) The resection depth reached the serosa. (H) Endoscopic follow-up 8 days after the procedure. (I) The rectal wound after resection of Pig 5. (J) The visible blood vessel was coagulated by using the snare. (K) The resection depth reached muscularis propria. (L) Endoscopic follow-up 8 days later.

All procedures were conducted by a skilled endoscopist (Y.L.). CERD-assisted resection was performed as follows (Figure 1). First, the cutting device was placed into the endoscopic channel and the external thread connection part was connected with the internal threaded connection part of the transparent cap (Figure 1A). The transparent cap was then attached to the forward-viewing endoscope. After the endoscope was inserted into the pig rectum (Figure 1B), the targeted rectal tissue was suctioned into the cap and gripped by tightening the snare (Figure 1C). Finally, the tissue was successfully resected (Figure 1D). If there was perforation, clips were used to close the wound (Figure 1E). If there was visible blood vessel of the wound (Figure 1I), the cutting device was used to coagulate the vessel (Figure 1J). All of the removed specimen was sent for pathological diagnosis and evaluation of resection depth (J.W.).

## Results

The targeted rectal tissue was successfully resected in all five pigs (Table 1). The average size of tissue was 10 mm. The wounds were at a distance of between 4 and 11 cm from the anus. The mean procedure time was 5.96 minutes, ranging from 2.5 to 10.0 minutes. CERD-assisted full-thickness resections were performed in Pigs 1 and 2. Each wound was completely closed by using three clips. In the other three pigs, the resection depth was muscularis propria. There was no intraoperative bleeding and no post-operative adverse events. All wounds healed with scar formation on the post-procedural 8th-day endoscopy follow-up.

**Table 1.** Basic information and evaluation indices when using a novel cap-assisted endoscopic resection device for rectal lesion resection

Pig no.	Tissue size, mm	Location (distance from the anus), cm	Technical success	Procedure time, min	Resection depth	Adverse events
1	12	5	Yes	10.0	Serosa	None
2	10	6	Yes	6.8	Serosa	None
3	6	4	Yes	2.5	Muscularis propria	None
4	12	9	Yes	4.7	Muscularis propria	None
5	10	11	Yes	5.8	Muscularis propria	None

## Discussion

During the mEMR-C procedure that we reported previously [1], a crescent-shaped electrosurgical snare needs to be passed through the sheath and precisely looped along the inner groove of the cap, which is time-consuming and difficult for inexperienced doctors. We previously proposed CERD to simplify the procedure of mEMR-C and initially explored the feasibility of the CERD for rectal tissue resection in an *in vitro* pig model [2]. In this pilot study, we modified CERD and demonstrated its safety and feasibility for rectal tissue resection *in vivo* in pigs for the first time.

The difference between the modified CERD and the previous version was the connection type of the metal snare of the transparent cap and the cutting device. In this version, the external thread connection part of the cutting device was tightly connected to the internal threaded connection part of the transparent cap, which helped the doctor pull the snare to resect and push the snare to coagulate.

As shown by our data, CERD-assisted resection is safe as no adverse events were observed. All five pigs were alive and the wounds healed by the 8th day after the procedure. Meanwhile, CERD has several advantages, including a high technical success rate and short procedure time. What is more, the resection depth was at least the muscularis propria.

Endoscopic submucosal resection with an endoscopic variceal ligation device (ESMR-L), similar to mEMR-C, was another relatively simple and safe procedure for rectal NETs of <10 mm. The difference between the two procedures is that ESMR-L has a ligator device to achieve band ligation below the tumor in order to achieve a high complete resection. Previous studies showed that ESMR-L enabled a deep vertical resection margin and a high complete resection rate of 93.1%–99.1% [3–6]. However, there was no randomized clinical trial comparing mEMR-C and ESMR-L for rectal NETs resection. In this animal study, we modified the transparent cap of the mEMR-C procedure and proved the safety and feasibility of CERD-assisted resection. For the treatment of rectal NETs that are <10 mm, further study should focus on comparing the safety and efficacy of CERD-assisted resection and ESMR-L to determine the better treatment.

This study also has several limitations. First, we resected the normal rectal tissues instead of the neuroendocrine tumor in the rectum, which did not completely simulate the procedure of rectal lesion resection in humans. Second, this study was designed as a single-arm study, with the absence of a comparison group. Third, the sample size was small. Therefore, clinical trials are still needed to confirm the safety and efficacy.

In conclusion, this pilot study demonstrated that CERD is safe and feasible for rectal tissue resection. Although it is not yet commercially available, the CERD might be a promising device for rectal NETs resection.

## Supplementary Material

goaf003_Supplementary_Data
