# Supplementary material for: The safety and feasibility of a novel cap-assisted endoscopic resection device for rectal tissue resection: a pilot study (with videos)
Source: Gastroenterol Rep (Oxf). 2025 Jan 30;13:goaf003. doi: 10.1093/gastro/goaf003 (PMC11783289; doi:10.1093/gastro/goaf003)
Supplement: goaf003_Supplementary_Data [file goaf003_supplementary_data.zip › ed7f6_Supplement data final version final verison 20241230.docx]

**Supplement Figure Legends**

**Supplementary figure 1**. Cap-assisted endoscopic resection device (CERD). (A) The inside view of the transparent cap of the older version of CERD. (B) The metal hook of the cutting device of the older version of CERD. (C) The physical image of the modified CERD. (D) The external thread connection part of the cutting part. (E) The inside view of the transparent cap (from the proximal part). (F) The outside view of the transparent cap (from the distal part).
