# Supplementary figures and images for: The safety and feasibility of a novel cap-assisted endoscopic resection device for rectal tissue resection: a pilot study (with videos)
Source: Gastroenterol Rep (Oxf). 2025 Jan 30;13:goaf003. doi: 10.1093/gastro/goaf003 (PMC11783289; doi:10.1093/gastro/goaf003)

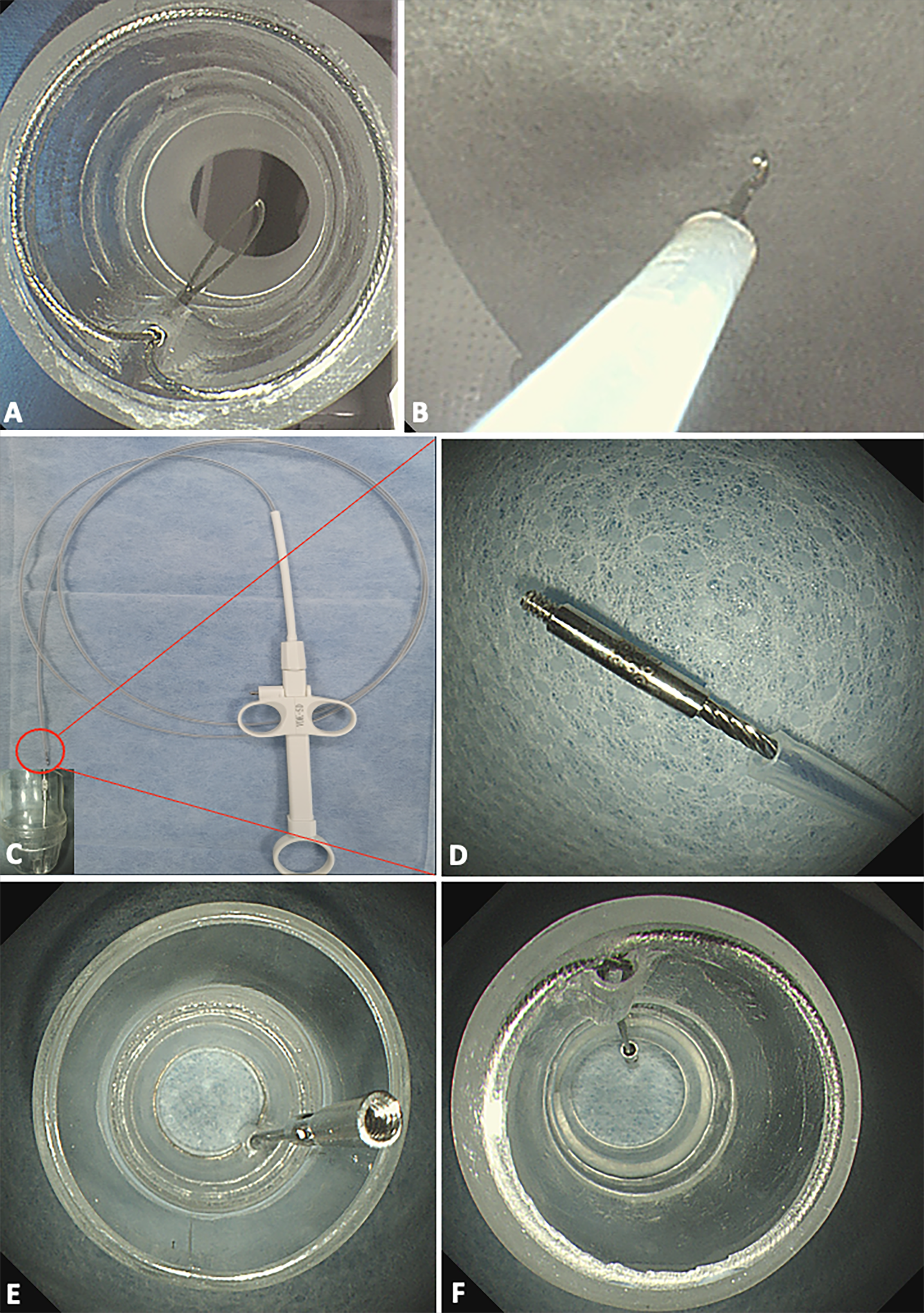

Supplement: goaf003_Supplementary_Data [file goaf003_supplementary_data.zip › 898ce_Supplementary Figure 1 final verison 20241230.tiff]
